# Tobacco Smoke-Induced Hepatic Injury with Steatosis, Inflammation, and Impairments in Insulin and Insulin-Like Growth Factor Signaling

**DOI:** 10.4172/2161-0681.1000269

**Published:** 2016-04-17

**Authors:** Suzanne M. de la Monte, M Tong, AR Agarwal, E Cadenas

**Affiliations:** 1 Liver Research Center, Department of Medicine, Rhode Island Hospital and the Alpert Medical School of Brown University, USA; 2 Division of Neuropathology and Departments of Pathology, Neurology, and Neurosurgery, Rhode Island Hospital and the Alpert Medical School of Brown University, USA; 3 Department of Pharmacology and Pharmaceutical Sciences, School of Pharmacy, University of Southern California, Los Angeles, CA, USA

**Keywords:** Tobacco, Cigarette smoke, Steatohepatitis, Mouse model, Insulin signaling

## Abstract

**Background:**

Alcoholic liver disease (ALD) is associated with impairments in hepatic insulin and insulin-like growth factor (IGF) signaling through cell growth, survival, and metabolic pathways. Since not all heavy drinkers develop ALD, co-factors may be important. Epidemiologic data indicate that most heavy drinkers smoke tobacco and experimental data revealed that low-level nitrosamine exposures, including those from tobacco, can cause steatohepatitis with hepatic insulin/IGF resistance and exacerbate ALD. We hypothesize that cigarette smoke (CS) exposures also cause liver injury with impaired hepatic insulin/IGF signaling, and thereby contribute to ALD.

**Methods:**

Adult male A/J mice were exposed to air for 8 weeks (A8), CS for 4 (CS4) or 8 (CS8) weeks, or CS for 8 weeks with 2 weeks recovery (CS8+R).

**Results:**

CS exposures caused progressive liver injury with disruption of the normal hepatic chord architecture, lobular inflammation, apoptosis or necrosis, micro-steatosis, sinusoidal dilatation, and nuclear pleomorphism. Histopathological liver injury scores increased significantly from A8 to CS4 and then further to CS8 (P<0.0001). The mean histological grade was also higher in CS8+R relative to A8 (P<0.0001) but lower than in CS4, reflecting partial resolution of injury by CS withdrawal. CS exposures impaired insulin and IGF-1 signaling through IRS-1, Akt, GSK-3β, and PRAS40. Livers from CS8+R mice had normalized or elevated levels of insulin receptor, pYpY-Insulin-R, 312S-IRS-1, 473S-Akt, S9-GSK-3β, and pT246-PRAS40 relative to A8, CS4, or CS8, reflecting partial recovery.

**Conclusion:**

CS-mediated liver injury and steatohepatitis with impairments in insulin/IGF signalling are reminiscent of the findings in ALD. Therefore, CS exposures (either first or second-hand) may serve as a co-factor in ALD. The persistence of several abnormalities following CS exposure cessation suggests that some aspects of CS-mediated hepatic metabolic dysfunction are not readily reversible.

## Introduction

Consequences of alcohol abuse and addiction are among the costliest healthcare problems in the world [1]. In the USA, alcohol abuse is the third leading preventable cause of death (88,000/year) [2–4], and on a global scale, it is a leading cause of liver related morbidity and mortality [1,5,6]. Although acute alcohol-related liver injury is typically reversible, some individuals develop steatohepatitis with chronic, progressive liver disease [7]. Acute alcoholic hepatitis is diagnosed according to standard clinical criteria together with histopathologic evidence of hepatic steatosis, acute inflammation, necrosis, ballooning degeneration of hepatocytes (keratin depletion), disorganization of the lobular architecture, mega-mitochondria, and accumulation of Mallory-Denk bodies [8,9] i.e., intracytoplasmic hyaline deposits corresponding to aggregated, misfolded, ubiquitinated proteins [10]. With progression to chronic alcoholic liver disease (ALD), hepatic function deteriorates due to multiple interrelated pathophysiological processes including: insulin resistance [11–13], cytotoxic and lipotoxic injury [14–17], inflammation [14,18] oxidative and ER stress [19–22] metabolic and mitochondrial dysfunction [23,24] decreased DNA synthesis [12,25,26] and increased cell death [15]. Progressive ALD is also marked by activation of pro-fibrogenic pathways [14] setting the stage for eventual development of cirrhosis and finally liver failure [24,27]. Increased understanding of ALD pathogenesis could help improve diagnostic and therapeutic approaches.

Variability in its development and progression suggests that co-factors contribute to the pathogenesis of ALD. One factor worthy of consideration is smoking since a very high percentage (~ 80%) of heavy drinkers/alcoholics abuse tobacco products, typically by cigarette smoking [28,29]. Although the co-carcinogenic effects of alcohol and tobacco have been well described [30–33] particularly with respect to the tobacco-specific nitrosamine, 4-(methylnitrosamino)-1-(3-pyridyl)-1-butanone (NNK) and its metabolites [30–34], little is known about non-carcinogenic, degenerative effects of smoking, apart from cardiovascular and pulmonary diseases. During the course of our investigations on mechanisms of liver insulin resistance, we discovered that chronic low level exposures to nitrosamines such as N-nitrodiethylamine (NDEA) cause steatohepatitis with insulin resistance, together with most other abnormalities present in ALD or non-alcoholic fatty liver disease (NAFLD) [35,36]. Moreover, we observed additive or synergistic effects of chronic high fat or alcohol feeding with NDEA exposures on insulin resistance degenerative diseases, including steatohepatitis [35–37].

Epidemiologic data reporting high rates of smoking among heavy drinkers suggest that the most significant co-factors in ALD development and progression are tobacco-specific nitrosamines. Since NNK is one of the most abundant nitrosamines present in tobacco smoke [38–41], initial studies examined the independent and additive effects of low-dose (sub-mutagenic) NNK and alcohol on steatohepatitis and associated molecular and biochemical abnormalities [42]. The results demonstrated that like other nitrosamines, NNK has toxic-degenerative effects on the liver, and causes steatohepatitis with hepatic insulin resistance and impaired signaling through PI3K-Akt. In addition, NNK exposures cause DNA damage, lipid peroxidation, pro-inflammatory cytokine activation, and ceramide accumulation in liver, and worsen the severity of ALD [42].

This study extends our research by addressing the most clinically and epidemiologically relevant question about the role of cigarette smoke (CS) exposure as a mediator of liver injury in adult male A/J mice [43,44]. In particular, the goal was to assess the degree to which smoking causes structural and functional abnormalities that correspond with effects of NNK and overlap with those of alcohol in the liver. Furthermore, we examined the effects of short-term smoking cessation on various parameters of liver disease. Although CS contains many other toxins and carcinogens, some of which can generate covalent adducts e.g. acetaldehyde and acrolein [45], there is no evidence that these compounds cause hepatic insulin resistance. In fact, acetaldehyde, the chief toxic metabolite of alcohol, causes inflammation, oxidative stress, DNA damage, mitochondrial dysfunction, and cell death, but not insulin resistance [46,47]. Analytical tools included histopathology, gene expression and multiplex enzyme-linked immunosorbent assays (ELISAs) of insulin/insulin-like growth factor, type 1 (IGF-1) signaling through the corresponding receptors, insulin receptor substrate (IRS) and Akt pathways, immunoassays of oxidative stress, and imaging mass spectrometry of hepatic lipid profiles.

## Experimental Section

### Experimental model

An experimental model of cigarette smoke (CS) exposure was generated in A/J adult male mice at 8 weeks of age. The subject groups (N=5/group) included controls that were exposed to air in chambers for 8 weeks (A8), CS for 4 (CS4) or 8 (CS8) weeks, or 8 weeks followed by 2 weeks recovery (CS+R). Research grade Kentucky 3R4F cigarettes (Tobacco Research Institute, University of Kentucky, Lexington, KY) were used to generate some in a Teague Enterprises, TE-10 Smoking Machine (Davis, CA), which is the industry standard [43,44]. Total particulate matter (TPM) per cigarette was 11 mg whereas nicotine was 0.73 mg. To simulate an environment rich in second-hand smoke, we used a mixture of side-stream and mainstream smoke in a ratio of 89% to 11%. The CS exposure regimen called for machine puffing of 6 cigarettes simultaneously, one time per minute for a total of 9 puffs. Mice in the CS4, CS8, and CS8+R groups were exposed to CS for 6 hours/day, 5 days/week. Continuous monitoring of the chambers confirmed that the atmosphere contained 21% oxygen and no more than 3 ppm of carbon monoxide. Mice were maintained under on a 12-hour light/dark cycle and they had free access to food and water. The University of Southern California’s Institutional Animal Care and Use Committee approved all experiments and protocols which conformed to guidelines established by the National Institutes of Health.

### Liver tissue collection and processing

Freshly harvested liver tissue was snap frozen and stored at −80°C for histopathological, biochemical, and molecular studies. For histological studies, 0.5 cm diameter samples of frozen liver were immersion-fixed in ice cold 4% neutral buffered paraformaldehyde containing 30% sucrose for cryoprotection. Tissue samples were then divided for paraffin embedding or cryosectioning. Paraffin-embedded histological sections (5 μm thick) were stained with Hematoxylin and Eosin (H&E) to assess the presence and severity of liver injury and hepatitis. Cryostat sections (10 microns) of fixed, non-paraffin embedded liver samples were stained with injury were graded using a standardized scoring system for non-alcoholic steatohepatitis [48–50].

### Hepatic lipid assays

Lipids were extracted from fresh frozen tissue with 2:1 chloroform-methanol [51]. Cholesterol and triglycerides were measured using the Analox GM7 Technology POCT Ltd, UK). Results were normalized to protein content in the samples.

### Insulin/IGF-1 signaling networks

We used bead-based multiplex ELISAs to measure immunoreactivity to the insulin receptor (IR), IGF-1 receptor (IGF-1R), IRS-1, Akt, proline-rich Akt substrate of 40 kDa (PRAS40), ribosomal protein S6 kinase (p70S6K), and glycogen synthase kinase 3β (GSK-3β), and pYpY1162/1163-IR, pYpY1135/1136-IGF-1R, pS312-IRS-1, pS473-Akt, pT246-PRAS40, pTpS421/424-p70S6K, and pS9-GSK-3β (Invitrogen, Carlsbad, CA). Samples (50 μg protein) were incubated with the beads, and captured antigens were detected with secondary antibodies and phycoerythrin-conjugated anti-rabbit IgG. Plates were read in a MAGPIX (Bio-Rad, Hercules, CA).

### mRNA expression studies

For gene expression studies, RNA was extracted using the RNA easy Mini Kit and cDNA templates were generated with the AMV 1st Strand cDNA Synthesis Kit (Hoffman-La Roche Ltd, UK). Duplex probe hydrolysis-based quantitative reverse transcriptase polymerase chain reaction (qRT-PCR) assays [37] were used to measure mRNA transcripts encoding insulin, IGF-1, and IGF-2 polypeptides, their corresponding receptors, and insulin receptor substrate (IRS) Types 1, 2 and 4 (Table 1). Probes were labeled with FAM and the internal control probe for β-actin was labeled with Y555. PCR amplifications were performed in a LightCycler 480 PCR machine and results were analyzed using the LightCycler Software 4.0.

### Statistics

Data depicted in graphs reflect group mean ± S.E.M. Data were analyzed by repeated GraphPad Prism 6 software (GraphPad Software, Inc., San Diego, CA).

## Results

### CS-induced hepatic injury

**Figure 1:**
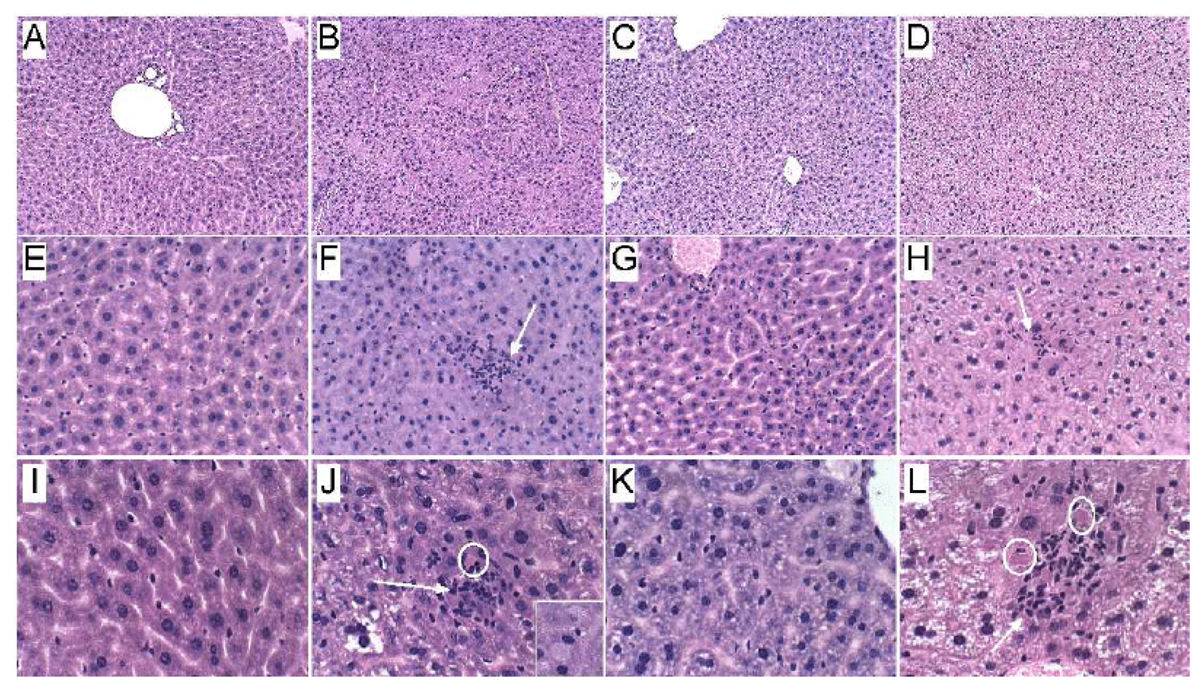
CS exposures alter hepatic architecture. Formalin-fixed, paraffin-embedded histological sections were stained with H&E to compare effects of CS exposures and withdrawal.

Histopathology: Formalin-fixed, paraffin-embedded H&E stained sections of livers revealed the normal uniform chord-like architecture in A8 controls. Livers from CS4 mice exhibited disruption of the normal chord-like architecture with marked variability in hepatocyte size and nuclear morphology, foci of necrosis, inflammation and cytoplasmic vacuolation, and scattered apoptotic (Councilman) bodies or hepatocytes with ballooning cytoplasmic degeneration. In CS8 livers, the hepatic chord architecture was partly restored and inflammation was milder and less aggregated compared with CS4 livers. However, the longer durations of CS exposure, including after 2 weeks recovery, resulted in hepatocellular microvesicular steatosis, which was more pronounced in the CS8+R group than CS8. Paradoxically, the CS8+R livers exhibited extreme cytoplasmic pallor, marked variability in nuclear size, small foci of inflammation, increased mitoses, and sinusoidal dilatation with congestion (Figure 1).

(A, E, I) In control livers, note the uniform chord-like architecture and cytology versus (B, F, J) the disorganized architecture, foci of lobular inflammation (arrow), necrosis (pale homogenous areas), apoptosis with cell loss (circles), and marked variability in nuclear size after 4 weeks of CS exposure, (C, G, K) steatohepatitis with foci of inflammation, diffuse microvesicular lipid droplets (small cytoplasmic vacuoles), apoptotic cells, foci of necrosis, cytoplasmic eosinophilia, and striking variation in hepatocyte size with many small nuclei after 8 weeks of CS exposure, and (D, H, L) markedly reduced nuclear density and extreme cytoplasmic pallor due to vacuolation (lipid accumulation) in CS8+R samples. Inset-ballooning degeneration. (Original magnifications, A–D=100x; E–H=200x; I–L=400x).

### Hepatic steatosis

**Figure 2:**
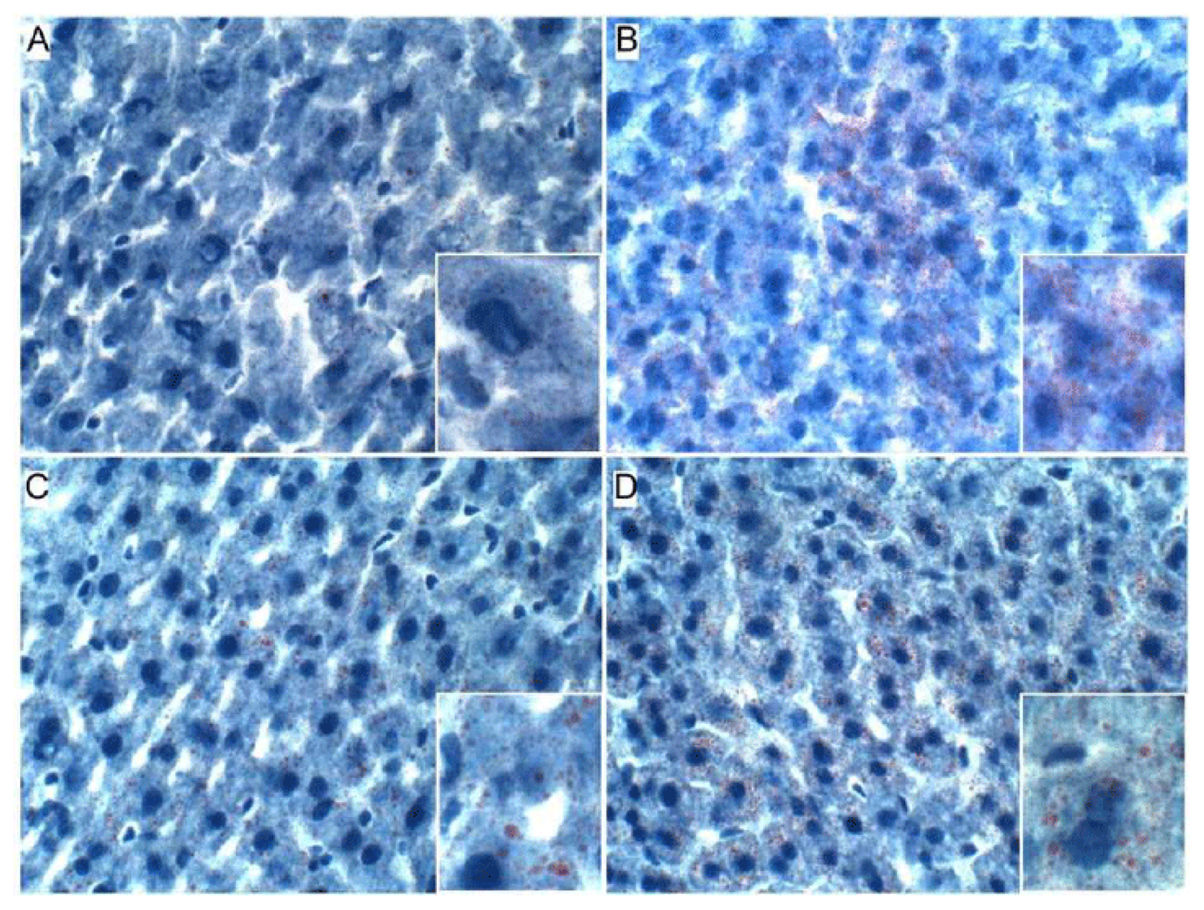
CS-induced lipid accumulation in hepatocytes. Cryostat sections of liver were stained with Oil Red O to detect neutral lipid (red droplets). Note minimal lipid accumulation in (A) control relative to (B) CS4. (C, D) Oil Red O staining is more diffuse and lower in density but coarser in quality (insets) after 8 weeks of CS exposure, with or without recovery. (Original magnification, 400x).

Oil red O staining revealed minimal or no detectable lipid in control hepatocytes, and small clusters of hepatocytes with abundant fine lipid droplets in CS4 livers. In both the CS8 and CS8+R livers, the intensity of hepatocellular cytoplasmic Oil red O staining was lower but more diffuse relative to CS4 livers. There was no clear difference between CS8 and CS8+R with respect to the density of Oil red O staining, despite different degrees of microsteatosis (Figure 2).

**Figure 3:**
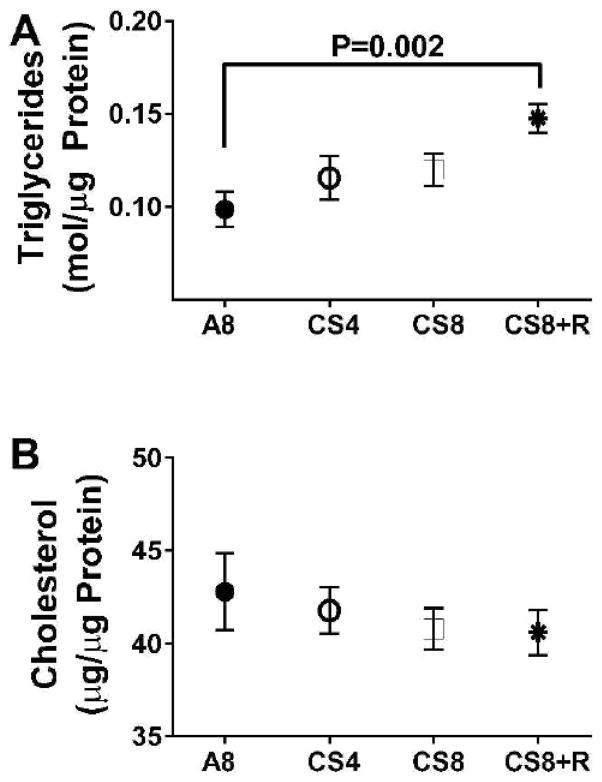
Hepatic triglyceride and cholesterol measurements. A) Triglyceride and B) cholesterol levels were measured using Analox reagents. Results were normalized to sample protein content. Inter-group differences were compared by one-way ANOVA with the Tukey post-test.

Correspondingly, hepatic triglyceride levels significantly differed among the groups (F=4.48; P=0.018) such that the levels were lowest in the control group, slightly higher in the CS4 and CS8 liver, and significantly elevated relative to control in the CS8+R group. Although hepatic cholesterol trended slightly downward from A8 to CS4, then CS8/CS8+R, the inter-group differences were not statistically significant (F=0.47, N.S.) (Figure 3).

### Pathological scoring of liver injury

Liver histopathology was graded using standardized scoring based on the presence and severity of hepatocellular steatosis, lobular inflammation, ballooning degeneration, and fibrosis [49] In addition, we systematically evaluated the specimens for other abnormalities associated with liver injury including loss of chord-like architecture, nuclear pleomorphism, apoptosis (individual cells; Councilman bodies), necrosis (micro-foci), and sinusoidal dilatation. Individual histopathological features were scored from 0 to 3 as described [49]. Total calculated scores including all features were also used for inter-group comparisons.

Among A8 controls, 1/5 had mild microvesicular steatosis and 2/5 had a single focus of lobular inflammation. None of the specimens exhibited ballooning degeneration or fibrosis and all had clear preservation of the chord-like architecture, uniform nuclear morphology, narrow sinusoids, and no evidence of apoptosis or necrosis. CS exposures increased the frequencies and degrees of steatosis, inflammation, loss of hepatic chord-like architecture, and foci of apoptosis and/or necrosis. Fibrosis was seldom observed and when present, it was mild, fine and focally distributed. In the CS4 group, 5/5 had mild lobular inflammation, 4/5 had mild microvesicular steatosis, 4/5 had foci of necrosis with apoptotic bodies and ballooning degeneration (3 moderate, 1 mild), and 1 had periportal fibrosis. In the CS8 group, 5/5 had moderate lobular inflammation and diffuses hepatic steatosis, 3/5 had micro-foci of necrosis, but no cases had fibrosis. Finally, in the CS8+R group, diffuse microsteatosis (5/5) and mild lobular inflammation (4/5) were the main abnormalities that persisted following recovery, whereas 0/5 cases exhibited necrosis, ballooning degeneration or fibrosis.

**Figure 4:**
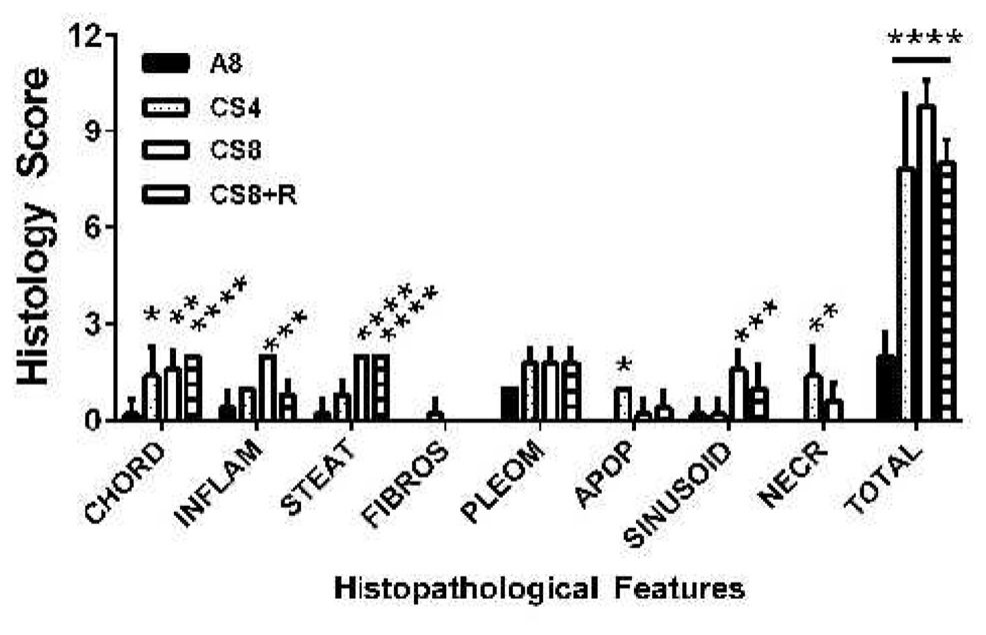
Comparative effects of CS exposures and withdrawal on liver histopathological scores. H&E stained sections of liver were scored (0–3) under code with respect to the extent or severity of: loss of the hepatic chord-like architecture (CHORD), lobular inflammation (INFLAM), microsteatosis (STEAT), chickenwire fibrosis (FIBROS), hepatocyte nuclear pleomorphism (PLEOM), apoptosis or councilman bodies (APOP), sinusoidal dilatation (SINUSOID), and foci of necrosis (NECR). In addition, the mean total scores (TOTAL) per sample were compared across the groups. Inter-group comparisons were made by 2-way ANOVA with post-hoc Tukey multiple comparison test. (*P<0.05; **P<0.01; ***P<0.001; ****P<0.0001).

Results of scoring the liver histology were analyzed by two-way ANOVA with post-hoc Tukey significance tests. The overall differences between A8 controls and the CS4, CS8 and CS8+R were all highly statistically significant (all P<0.0001). Further Tukey post hoc comparisons were made between A8 and the CS groups with respect to individual histopathological features. CS4 livers had significantly higher scores for loss of chord-like architecture and apoptosis relative to control (both P<0.05) (Figure 4).

CS8 livers had significantly higher scores for loss of chord-like architecture (P<0.01), lobular inflammation (P<0.0001), steatosis (P<0.0001), sinusoidal dilatation (P<0.001), and necrosis (P<0.01). CS8+R livers had significantly higher scores for loss of the chord-like architecture (P<0.0001) and steatosis (P<0.0001), but not sinusoidal dilation, necrosis, or apoptosis. Total histopathological scores (mean ± S.E.M.) increased from A8 (2.0 ± 0.31), to CS4 (7.8+1.07), to CS8 (9.8±0.37), and then declined in the CS8+R group (8.0 ± 0.32). The differences between A8 and each of the CS groups were highly statistically significant (P<0.0001). In addition, the mean total score was significantly higher in CS8 compared with CS4 (P<0.0001), whereas the differences between CS4 and CS8+R were not statistically significant. The latter suggests that the short period of recovery from CS reduced the severity of liver injury in the CS8 exposure group.

### Modulation of insulin/IGF upstream mRNA expression

Hepatic mRNA levels of insulin, IGF-1, IGF-2 polypeptides, their corresponding receptors, and IRS-1, IRS-2, and IRS-4 were measured by probe-hydrolysis based duplex qRT-PCR analysis with β-actin co-amplified as the internal control gene. Inter-group statistical comparisons were made using one-way ANOVA tests (Table 2); the post-hoc repeated measures Tukey test results are shown.

**Figure 5:**
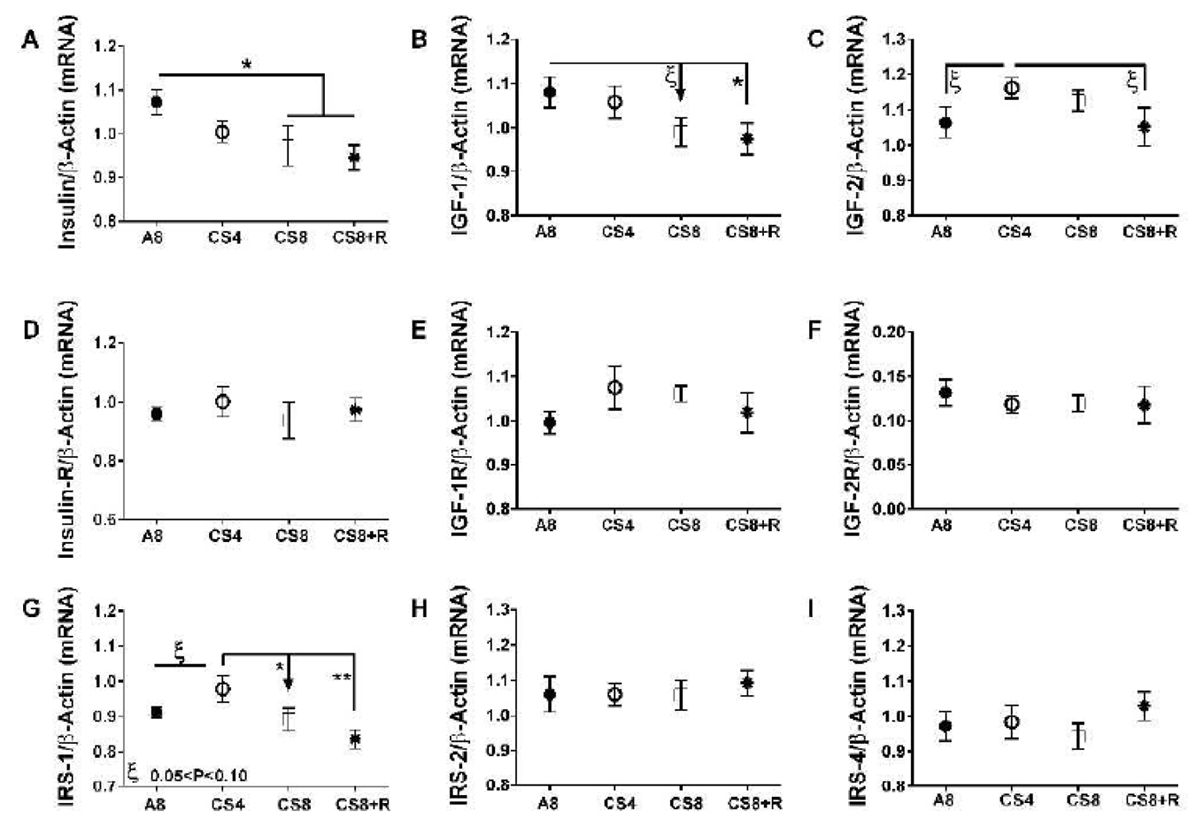
Effects of CS on insulin/IGF/IRS signaling-related gene expression. RNA was reverse transcribed and the cDNAs were used in duplex qPCR reactions to measure A) insulin, B) IGF-1, C) IGF-2, D) insulin receptor, E) IGF-1 receptor, F) IGF-2 receptor, G) IRS-1, H) IRS-2, and I) IRS-4 mRNA transcripts with gene expression normalized to simultaneously measured β-actin. Data were analyzed by 1-way ANOVA (Table 1) and the post-hoc Tukey multiple comparison test (*P<0.05; **P<0.01, ξ − 0.05<P<0.10).

Insulin and IGF-1 polypeptide mRNA levels declined with increasing duration of smoke exposure, and those trends were not abrogated by 2-weeks CS recovery. Correspondingly, the CS8 and CS8+R livers had similarly low expression of insulin and IGF-1 polypeptide genes. IGF-2 polypeptide expression was slightly but not significantly higher in CS4 and CS8 livers relative to control and CS8+R. CS ± R had no significant effect on insulin receptor, IGF-1 receptor, and IGF-2 receptor gene expression. Hepatic IRS-1 expression was highest in CS4 and lowest in CS8+R livers; the differences between A8 or CS8+R and CS4 reached a statistical trend (0.05<P<0.10). CS had no effect on significant or trend effects hepatic IRS-2 or IRS-4 gene expression (Figure 5).

Results of one-way ANOVA tests comparing the mean mRNA levels of insulin, IGF-1, IGF-2, their corresponding receptors, IRS-1, IRS-2, and IRS-4 in livers from A8 control, CS4, CS8, and CS8+R mice. Italicized values reflect statistical trends, i.e. 0.05<P<0.10.

### Insulin/IGF-1 signaling through IRS-1

Hepatic immunoreactivity corresponding to total protein and phospho-protein levels of the insulin receptor (Insulin R), IGF-1R, and IRS-1 using multi-plex ELISAs, and relative levels of protein phosphorylation were calculated (phospho/total). Inter-group statistical comparisons were made using one-way ANOVA tests (Table 3); the post-hoc repeated measures Tukey test results are shown.

### Upstream signaling protein expression

Insulin receptor protein expression was lowest in CS4 livers, and similar in A8, CS8 and CS8+R livers. The CS4-associated reduction in Insulin R immunoreactivity was significant relative to the CS8 group (P<0.05). IGF-1 receptor expression was significantly reduced in all 3 CS groups relative to control. The mean levels of IRS-1 immunoreactivity were similar across all groups.

### Upstream signaling protein phosphorylation

**Figure 6:**
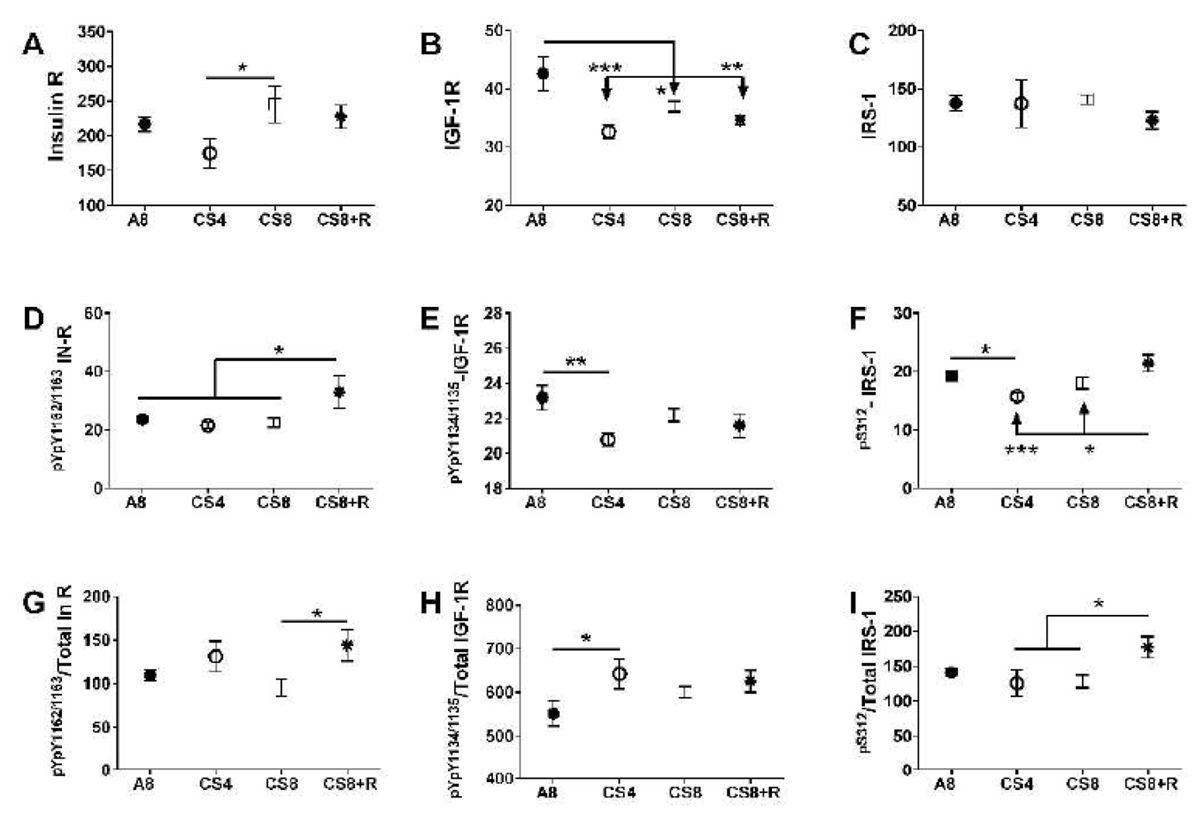
CS exposure alters insulin/IGF-IRS-1 signaling. Liver protein homogenates were used in bead-based multiplex ELISAs to measure immunoreactivity to: A) insulin receptor (R), B) IGF-1R, C) IRS-1, D) pYpY1162/1163-InsR, E) pYpY1135/1136-IGF-1R, and F) pS312-IRS-1. Relative phosphorylation is represented by the calculated ratios of: G) pYpY1162/1163-/total Insulin R, H) pYpY1135/1136-/total IGF-1R, and I) pS312-/total IRS-1. Inter-group comparisons were made by 1-way ANOVA tests (Table 1). Post-hoc Tukey multiple comparison test results are depicted in the graphs (*P<0.05; **P<0.01; ***P<0.001).

The effects of CS on insulin receptor, IGF-1 receptor, and IRS-1 phosphorylation were varied. Insulin receptor tyrosine phosphorylation was increased in CS8+R relative to A8, CS4, and CS8. Tyrosine phosphorylation of the IGF-1 receptor was highest in A8 levers and similarly reduced in the CS groups, although only the A8 vs CS4 group difference was significant (P<0.01). S312 phosphorylation of IRS-1 was reduced by CS4 and CS8 relative to A8 and/or CS8+R, while the levels in the A8 and CS8+R groups were similar (Figure 6).

### Relative phosphorylation of upstream proteins

Relative tyrosine phosphorylation of the insulin receptor was similar across the 4 groups; however, the slightly increased levels of total insulin receptor protein expression in the CS8 group rendered the difference between CS8 and CS8+R statistically significant (P<0.05). Similarly, the mean relative levels of IGF-1 receptor tyrosine phosphorylation were similarly elevated in the CS groups, but the somewhat larger decline in phosphorylated IGF-1R, rendered the A8 vs CS4 difference significant (P<0.05). The reductions in relative S312-phosphorylation of IRS-1 in the CS4 and CS8 groups relative to CS8+R are largely due to the reduced hepatic levels of 312S-IRS-1.

### Akt pathway activation

Signaling downstream through Akt was examined to assess the impact of CS exposure on mediators of cell survival, growth, and metabolism. Hepatic immunoreactivity corresponding to total protein and phospho-protein levels of the Akt, GSK-3β, p70S6K, and PRAS40 were measured using multi-plex Akt Pathway ELISAs, and the relative levels of protein phosphorylation (phospho/total) were calculated. Inter-group statistical comparisons were made using one-way ANOVA tests (Table 3). Post-hoc repeated measures Tukey test results are shown.

Results of one-way ANOVA tests (F-Ratios and P-Values) comparing A8 control, CS4, CS8, and CS8+R mean total protein, phospho-protein, and calculated relative levels of protein phosphorylation as assessed using multiplex total and phospho-Akt pathway reagents (see Methods). Italicized values reflect statistical trends, i.e. 0.05<P<0.10.

### Downstream Akt pathway proteins

Akt and GSK-3β protein levels significantly differed among the groups (Table 3). Akt expression was significantly reduced in all CS groups relative to control, and the mean level in CS4 was significantly lower than in the other 3 groups. GSK-3β immunoreactivity was significantly lower in CS4 and CS8+R relative to A8 and CS8. In contrast, the mean levels of PRAS40 and p70S6K were similar across the 4 groups.

### Akt pathway protein phosphorylation and relative phosphorylation

**Figure 7:**
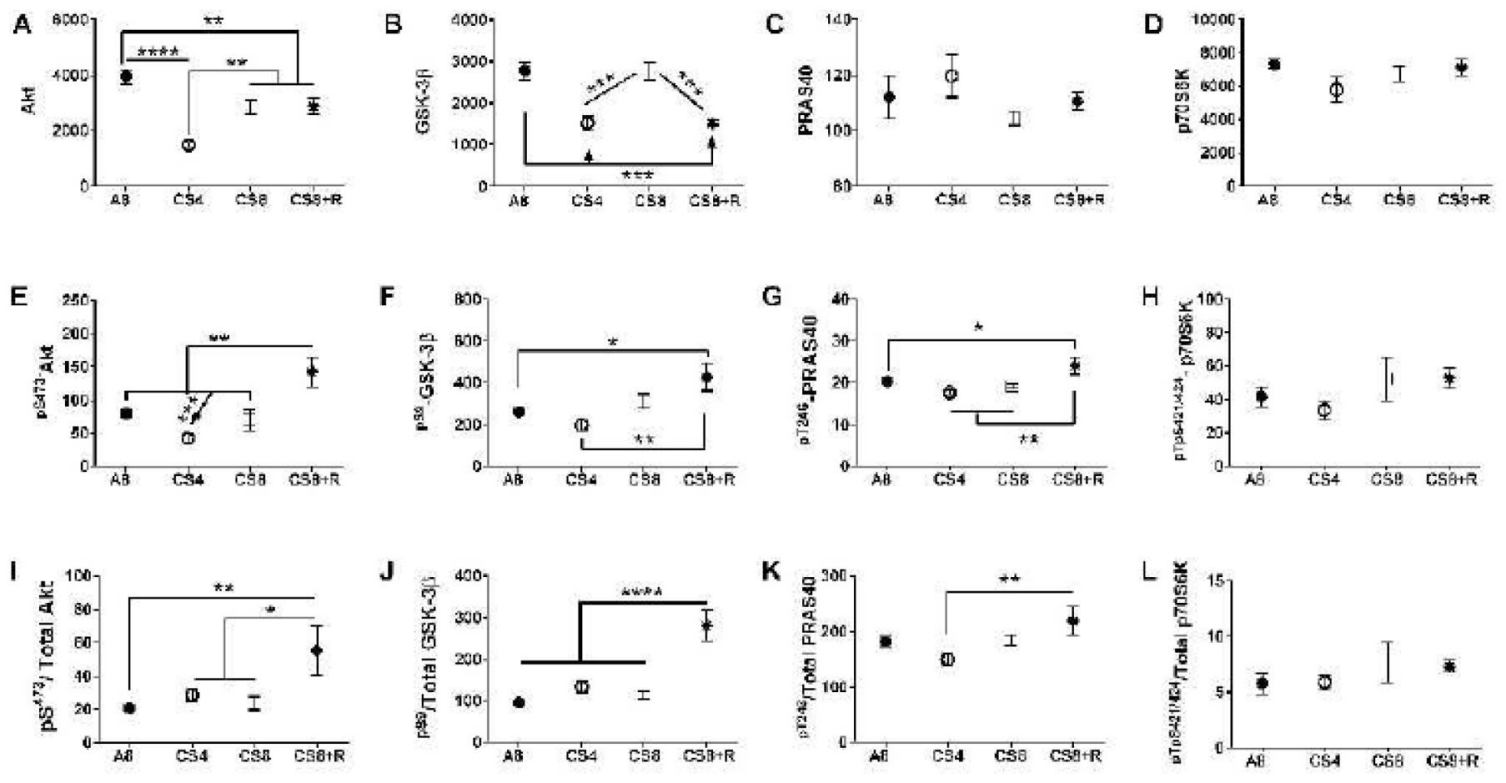
CS exposure effects on Akt pathways. Liver protein homogenates were used in bead-based multiplex ELISAs to measure immunoreactivity to: A) Akt, B) GSK-3β, C) PRAS40, D) p70S6K, E) pS473 AKT, F) pS9-GSK-3β, G) pT246-PRAS-40, and H) pTpS421/424-p70S6K. Relative phosphorylation is represented by the calculated ratios of: I) pS473-/total AKT, J) pS9-/total GSK-3β, K) pT246-/total PRAS40, and L) pTpS421/424-/total p70S6K. Data were analyzed by 1-way ANOVA (Table 1). Post-hoc Tukey multiple comparison test results are depicted in the graphs (*P<0.05; **P<0.01; ***P<0.001; ****P<0.0001).

The mean levels of pS473-AKT and pS473-AKT/total Akt were significantly elevated in the CS8+R livers relative to the other three groups. While total GSK-3β protein was suppressed in the CS4 and CS8+R groups relative to control and CS8, the levels of pS9-GSK-3β and pS9-GSK-3β/total GSK-3β were significantly elevated in the CS8+R group, corresponding with its effects on Akt activation. pT246-PRAS-40 expression was modestly but significantly elevated in the CS8+R group, while pT246-/total PRAS40 was modestly suppressed in the CS4 group. These results suggest that the 2-week recovery from 8 weeks of CS exposure enhanced hepatic signaling through crucial trophic factor regulated cell growth, survival and metabolism. In contrast, no significant inter-group differences or trends occurred with p70S6K. pTpS421/424-p70S6K, or pTpS421/424-p70S6K/total p70S6K (Figure 7).

## Discussion

The pathophysiological effects of inhaling CS are caused by exposure to numerous toxins present in tobacco smoke. Both volatile and non-volatile toxins, in addition to tobacco-specific nitrosamines represent key mediators of tissue injury. The two most abundant and toxic tobacco-specific nitrosamines in CS are 4-(methylnitrosamino)-1-(3-pyridyl)-1-butanone (NNK) and N′-nitrosonornicotine (NNN) [41,52,53]. There are between 1 μg and 9 μg of tobacco-specific nitrosamines present in each cigarette, and there can be up to 8 μg of other types of nitrosamines. Importantly, up to 2 μg of nitrosamine products can be released into air, leading to significant second-hand exposures [54]. For decades up to the present, the overwhelming body of research conducted on nitrosamine-induced diseases was devoted to carcinogenesis. However, there is now growing evidence indicating that low-level nitrosamine exposures can also compromise health and promote degenerative tissue injury mediated by inflammation, disrupted insulin/IGF signaling mechanisms through cell survival and metabolic pathways, increased oxidative stress, and altered lipid metabolism [36,55,56]. Previously, we showed that NNK and NDEA can cause steatohepatitis and exacerbate alcohol or chronic high fat diet induced liver injury [36,42]. The present work is the first to characterize histopathological, molecular, and biochemical effects of CS exposures on the liver.

The histopathological studies revealed that CS exposures caused liver injury that was associated with features typically present in steatohepatitis due to other causes. In this regard, the CS-exposed livers exhibited increased inflammation, necrosis, apoptosis, increased cell turnover, disorganization of the lobular architecture and steatosis. We did not observe increased fibrosis on H&E stained sections, but most likely the CS exposures were not sufficiently prolonged to activate fibrogenesis pathways in liver. Similarly, 8 weeks of side stream CS exposure is not sufficient to cause pulmonary fibrosis or emphysema [43,44,57]. CS-mediated inflammation and hepatocellular injury-related steatohepatitis scores increased in the CS4 group and further in the CS8 group, whereas relatively muted responses were observed in CS8+R livers, reflecting beneficial effects of CS withdrawal. In contrast, the highest levels of necrosis, apoptotic bodies, and nuclear pleomorphism (reflecting increased cell turnover) occurred in CS4 livers. The relatively lower levels of active hepatocyte injury in the CS8 and CS8+R samples could reflect adaptive responses to chronic CS exposure.

Although these studies do not exclude potential contributions of acetaldehyde in the CS, which causes multi-molecular adducts and liver injury [46], the fact that NNK itself causes steatohepatitis [42] supports our hypothesis that at least one of the hepatotoxic agents in CS is a nitrosamine, and probably NNK and/or NNN. Mechanistically, NNK and CS exposures lead to NNK-mediated O6-methyguanine adducts in liver [42,58]. At high levels NNK is mutagenic/carcinogenic [59,60], but at low levels NNK causes steatohepatitis and hepatic insulin/IGF resistance with dysregulated lipid metabolism [42].

Besides NNK or NNN, other potential mediators of CS-associated liver injury include carbon monoxide (CO) exposure, acetaldehyde adduct formation, and acrolein-induced hepatotoxicity. In general, CO toxicity leads to hypoxic-ischemic injury and cell death [61]. However, since the CO level in the exposure chambers was low (3 ppm), it is highly unlikely that the histopathological, molecular, and biochemical abnormalities observed after CS exposure would be attributable to toxic effects of CO. Acetaldehyde is present in CS and can cause liver injury due to protein and DNA adduct formation [46,62]. However, since acetaldehyde mainly causes oxidative stress and cytotoxic injury without impairing insulin/IGF signaling [47], it is unlikely that acetaldehyde adduct accumulation was the main cause of liver pathology and dysfunction. Finally, acrolein is present in CS and highly reactive producing toxic effects on mitochondria [63]. However, acrolein toxicity mainly occurs at the initial points of contact such as the respiratory and gastrointestinal tracts. Therefore, liver is not likely to be a major *in vivo* target of acrolein toxicity and correspondingly, the studies showing adverse effects on hepatocytes were conducted *in vitro*.

Several significant abnormalities pertaining to insulin/IGF signaling, including insulin and IGF-1 receptor, Akt, pYpY1134/1135-IGF-1R, pS312-IRS-1, and GSK-3β protein expression in CS4 livers either normalized or trended back toward control levels in the CS8 livers. These effects may account for some of the apparent improvements in liver histology from CS4 to CS8. On the other hand, several of the CS4 abnormalities were worsened by longer durations of CS exposure, including inhibition of insulin, IGF-1, and IRS-1 mRNA expression, suggesting that molecular changes in gene expression become established with longer durations of CS exposure, and possibly triggering a course of progressive liver degeneration. In this regard, it is noteworthy that after 2-weeks of CS recovery after 8 weeks exposure, although a number of indices, such as pYpY1161/1162-insulin receptor, pS473-Akt, pS9-GSK-3β, and pT246-PRAS40 improved significantly relative to CS4 and/or CS8, reflecting enhanced insulin signaling through Akt pathways, other indices were either unchanged or worsened. For example, expression of insulin, IGF-1, and IRS-1 mRNAs declined, insulin and IGF-1 protein expression were the same as in CS8, and pS312-IRS-1 and pS312/total IRS-1, which inhibit IRS-1 signaling, were increased. Furthermore, hepatic steatosis (histologic and triglyceride levels) and injury/repair (apoptosis/nuclear mitoses) were more pronounced in CS8+R than in CS8. One potential explanation for this phenomenon is that the cascade of liver injury is established after 8 weeks continues to progress even in the absence of further CS exposure.

## Conclusion

These studies demonstrate that CS exposures cause progressive liver injury with impairments in hepatic insulin/IGF signaling that overlap with features of ALD. Importantly, CS-associated impairments in hepatic insulin/IGF signaling were evident at the receptor level and manifested by alterations in receptor protein expression and phosphorylation. With longer periods of CS exposure, the abnormalities progressed due to inhibition of insulin, IGF-1, and IRS-1 gene expression. The findings suggest that early responses to CS exposures are associated with compensatory enhancements in signaling through cell growth, survival, and metabolic pathways, but the long-term effects of CS exposure may not be entirely reversible. These results provide a mechanism by which CS exposures could exacerbate alcoholic liver disease.

## Figures and Tables

**Table 1:** PCR Primer Pairs and Probes used for Duplex qRT-PCR Analysis.

mRNA	UPL primers	Sequence 5′-3′	Amplicon	Position	Probe ID#
	mINS-UPL-For	GGCATTGTGGATCAGTGCT	75bp	NM_008386.3	32
Insulin	mINS-UPL-Rev	AGGTGGGCCTTAGTTGCAG		445-519	
	mINR-UPL-For	GCCAAGTAGCATCATGTCCA	73bp	NM_010568.2	52
Insulin Receptor	mINR-UPL-Rev	ACATGCAGAACAATTTCAGTCC		9225-9297	
	mIGF1-UPL-For	ACATAGGTGGCCTTCTCCAG	60bp	NM_010512.4	4
IGF-1	mIGF1-UPL-Rev	TGGAATCACCAAATCCAACC		3411-3470	
	mIGF1R-UPL-For	GTGCTCTGCCTCTGTCCAC	65bp	NM_010513.2	84
IGF-1 Receptor	mIGF1R-UPL-Rev	TGGAGTTGAGGATTGCACTG		8234-8298	
	mIGF2-UPL-For	CGTCCCTCAGTGTCATCTTG	69bp	NM_010514.3	73
IGF-2	mIGF2-UPL-Rev	TGTAGAGCTCCAGACCTCCTG		3096-3164	
	mIGF2R-UPL-For	CCTTCTCTAGTGGATTGTCAAGTG	62bp	NM_010515.2	33
IGF-2 Receptor	mIGF2R-UPL-Rev	AGGGCGCTCAAGTCATACTC		3387-3448	
Insulin Receptor Substrate, Type 1	mIRS1-UPL-For mIRS1-UPL-Rev	GAGGAGCAACACTCTACTTCTGG AACCCTTGCTCCTTCCTTG	70bp	NM_010570.4 6275-6344	79
Insulin Receptor Substrate, Type 2	mIRS2-UPL-For mIRS2-UPL-Rev	GGTTTCATGTCCCTTGACGA CTGTGGCTACTGAAGGCTCTC	65bp	NM_001081212.1 1477-1541	19
Insulin Receptor	mIRS4-UPL-For	GTCTGCAAACGCGGCTAC	61bp	NM_010572.2	38

**Table 2:** ANOVA Results of mRNA Studies.

mRNA	F-Ratio	P-Value
Insulin	2.83	0.071
IGF-1	2.15	N.S.
IGF-2	1.67	N.S.
Insulin Receptor	0.32	N.S.
IGF-1 Receptor	1.00	N.S.
IGF-2 Receptor	0.21	N.S.
IRS-1	4.05	0.025
IRS-2	0.17	N.S.
IRS-4	0.07	N.S.

**Table 3:** Multiplex ELISA Results-ANOVA.

Protein	F-Ratio	P-Value
Insulin Receptor	2.38	0.107
IGF-1 Receptor	6.46	0.004
IRS-1	0.48	N.S.
Akt	16.58	<0.0001
GSK-3β	17.53	<0.0001
p70S6K	1.72	N.S.
PRAS40	1.18	N.S.
Phospho-Protein	F-Ratio	P-Value
pYpY1162/1163-Insulin R	3.17	0.050
pYpY1135/1136-IGF-1R	3.26	0.049
pS312-IRS-1	5.67	0.008
pS473-Akt	8.71	0.001
pS9-GSK-3β	5.93	0.006
pTpS421/424-p70S6K	1.23	N.S.
Phospho/Total Protein	F-Ratio	P-Value
pYpY1162/1163-Insulin R/Insulin R	2.46	0.10
pYpY1135/1136-IGF-1R/IGF-1R	2.31	N.S.
pS312-IRS-1/IRS-1	3.05	0.05
pS473-Akt/Akt	4.07	0.02
pS9-GSK-3β/GSK-3β	16.61	<0.0001
pTpS421/424-p70S6K/p70S6K	0.77	N.S.

## References

[1] Paula H, Asrani SK, Boetticher NC, Pedersen R, Shah VH (2010). Alcoholic liver disease-related mortality in the United States: 1980–2003. Am J Gastroenterol.

[2] Serdula MK, Brewer RD, Gillespie C, Denny CH, Mokdad A (2004). Trends in alcohol use and binge drinking, 1985–1999: results of a multistate survey. Am J Prev Med.

[3] Mokdad AH, Giles WH, Bowman BA, Mensah GA, Ford ES (2004). Changes in health behaviors among older Americans, 1990 to 2000. Public Health Rep.

[4] Centers for Disease Control and Prevention (CDC) (2008). Alcohol-attributable deaths and years of potential life lost among American Indians and Alaska Natives--United States, 2001–2005. MMWR Morb Mortal Wkly Rep.

[5] Miller AM, Horiguchi N, Jeong WI, Radaeva S, Gao B (2011). Molecular mechanisms of alcoholic liver disease: innate immunity and cytokines. Alcohol Clin Exp Res.

[6] McCullough AJ, O’Shea RS, Dasarathy S (2011). Diagnosis and management of alcoholic liver disease. J Dig Dis.

[7] O’Shea RS, Dasarathy S, McCullough AJ, Practice Guideline Committee of the American Association for the Study of Liver Diseases; Practice Parameters Committee of the American College of Gastroenterology (2010). Alcoholic liver disease. Hepatology.

[8] Tannapfel A, Denk H, Dienes HP, Langner C, Schirmacher P (2011). Histopathological diagnosis of non-alcoholic and alcoholic fatty liver disease. Virchows Arch.

[9] Yerian L (2011). Histopathological evaluation of fatty and alcoholic liver diseases. J Dig Dis.

[10] Bardag-Gorce F (2010). Effects of ethanol on the proteasome interacting proteins. World J Gastroenterol.

[11] de la Monte SM, Yeon JE, Tong M, Longato L, Chaudhry R (2008). Insulin resistance in experimental alcohol-induced liver disease. J Gastroenterol Hepatol.

[12] Pang M, de la Monte SM, Longato L, Tong M, He J (2009). PPARdelta agonist attenuates alcohol-induced hepatic insulin resistance and improves liver injury and repair. J Hepatol.

[13] Longato L, Ripp K, Setshedi M, Dostalek M, Akhlaghi F (2012). Insulin resistance, ceramide accumulation, and endoplasmic reticulum stress in human chronic alcohol-related liver disease. Oxid Med Cell Longev.

[14] Cohen JI, Nagy LE (2011). Pathogenesis of alcoholic liver disease: interactions between parenchymal and non-parenchymal cells. J Dig Dis.

[15] Derdak Z, Lang CH, Villegas KA, Tong M, Mark NM (2011). Activation of p53 enhances apoptosis and insulin resistance in a rat model of alcoholic liver disease. J Hepatol.

[16] McVicker BL, Tuma DJ, Kubik JL, Tuma PL, Casey CA (2006). Ethanol-induced apoptosis in polarized hepatic cells possibly through regulation of the Fas pathway. Alcohol Clin Exp Res.

[17] de la Monte SM, Longato L, Tong M, DeNucci S, Wands JR (2009). The liver-brain axis of alcohol-mediated neurodegeneration: role of toxic lipids. Int J Environ Res Public Health.

[18] Ronis MJ, Butura A, Korourian S, Shankar K, Simpson P (2008). Cytokine and chemokine expression associated with steatohepatitis and hepatocyte proliferation in rats fed ethanol via total enteral nutrition. Exp Biol Med (Maywood).

[19] Malhi H, Kaufman RJ (2011). Endoplasmic reticulum stress in liver disease. J Hepatol.

[20] Pandol SJ, Gorelick FS, Gerloff A, Lugea A (2010). Alcohol abuse, endoplasmic reticulum stress and pancreatitis. Dig Dis.

[21] Feldstein AE, Bailey SM (2011). Emerging role of redox dysregulation in alcoholic and nonalcoholic fatty liver disease. Antioxid Redox Signal.

[22] Kaplowitz N, Ji C (2006). Unfolding new mechanisms of alcoholic liver disease in the endoplasmic reticulum. J Gastroenterol Hepatol.

[23] Ding WX, Manley S, Ni HM (2011). The emerging role of autophagy in alcoholic liver disease. Exp Biol Med (Maywood).

[24] Purohit V, Gao B, Song BJ (2009). Molecular mechanisms of alcoholic fatty liver. Alcohol Clin Exp Res.

[25] Banerjee K, Mohr L, Wands JR, de la Monte SM (1998). Ethanol inhibition of insulin signaling in hepatocellular carcinoma cells. Alcohol Clin Exp Res.

[26] Sasaki Y, Hayashi N, Ito T, Fusamoto H, Kamada T (1994). Influence of ethanol on insulin receptor substrate-1-mediated signal transduction during rat liver regeneration. Alcohol Alcohol.

[27] Vidali M, Stewart SF, Albano E (2008). Interplay between oxidative stress and immunity in the progression of alcohol-mediated liver injury. Trends Mol Med.

[28] Romberger DJ, Grant K (2004). Alcohol consumption and smoking status: the role of smoking cessation. Biomed Pharmacother.

[29] Kalman D, Kim S, DiGirolamo G, Smelson D, Ziedonis D (2010). Addressing tobacco use disorder in smokers in early remission from alcohol dependence: the case for integrating smoking cessation services in substance use disorder treatment programs. Clin Psychol Rev.

[30] de Boer MF, Sanderson RJ, Damhuis RA, Meeuwis CA, Knegt PP (1997). The effects of alcohol and smoking upon the age, anatomic sites and stage in the development of cancer of the oral cavity and oropharynx in females in the south west Netherlands. Eur Arch Otorhinolaryngol.

[31] Duell EJ (2012). Epidemiology and potential mechanisms of tobacco smoking and heavy alcohol consumption in pancreatic cancer. Mol Carcinog.

[32] Johnson NW, Warnakulasuriy S, Tavassoli M (1996). Hereditary and environmental risk factors; clinical and laboratory risk matters for head and neck, especially oral, cancer and precancer. Eur J Cancer Prev.

[33] Tramacere I, Negri E, Bagnardi V, Garavello W, Rota M (2010). A meta-analysis of alcohol drinking and oral and pharyngeal cancers. Part 1: overall results and dose-risk relation. Oral Oncol.

[34] Go VL, Gukovskaya A, Pandol SJ (2005). Alcohol and pancreatic cancer. Alcohol.

[35] Tong M, Longato L, de la Monte SM (2010). Early limited nitrosamine exposures exacerbate high fat diet-mediated type 2 diabetes and neurodegeneration. BMC Endocr Disord.

[36] Tong M, Neusner A, Longato L, Lawton M, Wands JR (2009). Nitrosamine exposure causes insulin resistance diseases: relevance to type 2 diabetes mellitus, non-alcoholic steatohepatitis, and Alzheimer’s disease. J Alzheimers Dis.

[37] Andreani T, Tong M, de la Monte SM (2014). Hotdogs and Beer: Dietary Nitrosamine Exposure Exacerbates Neurodevelopmental Effects of Ethanol in Fetal Alcohol Spectrum Disorder. JDAR.

[38] Trushin N, Hecht SS (1999). Stereoselective metabolism of nicotine and tobacco-specific N-nitrosamines to 4-hydroxy-4-(3-pyridyl)butanoic acid in rats. Chem Res Toxicol.

[39] Hoffmann D, Hecht SS, Ornaf RM, Wynder EL (1974). N′-nitrosonornicotine in tobacco. Science.

[40] Hecht SS, Ornaf RM, Hoffmann D (1975). Chemical studies on tobacco smoke. XXXIII. N′-nitrosonornicotine in tobacco: analysis of possible contributing factors and biologic implications. J Natl Cancer Inst.

[41] Stepanov I, Feuer R, Jensen J, Hatsukami D, Hecht SS (2006). Mass spectrometric quantitation of nicotine, cotinine, and 4-(methylnitrosamino)-1-(3-pyridyl)-1-butanol in human toenails. Cancer Epidemiol Biomarkers Prev.

[42] Zabala V, Tong M, Yu R, Ramirez T, Yalcin EB (2015). Potential contributions of the tobacco nicotine-derived nitrosamine ketone (NNK) in the pathogenesis of steatohepatitis in a chronic plus binge rat model of alcoholic liver disease. Alcohol Alcohol.

[43] Agarwal AR, Yin F, Cadenas E (2014). Short-term cigarette smoke exposure leads to metabolic alterations in lung alveolar cells. Am J Respir Cell Mol Biol.

[44] Agarwal AR, Zhao L, Sancheti H, Sundar IK, Rahman I (2012). Short-term cigarette smoke exposure induces reversible changes in energy metabolism and cellular redox status independent of inflammatory responses in mouse lungs. Am J Physiol Lung Cell Mol Physiol.

[45] Salaspuro M (2011). Acetaldehyde and gastric cancer. J Dig Dis.

[46] Setshedi M, Wands JR, Monte SM (2010). Acetaldehyde adducts in alcoholic liver disease. Oxid Med Cell Longev.

[47] Tong M, Longato L, Nguyen QG, Chen W, Spaisman A (2011). Acetaldehyde-mediated neurotoxicity: relevance to fetal alcohol spectrum disorders. Oxid Med Cell Long.

[48] Ramirez T, Tong M, de la Monte SM (2014). Chronc-Binge Model of Alcoholic Hepatitis in Long Evans Rats. J Drug Alc Res.

[49] Kleiner DE, Brunt EM (2012). Nonalcoholic fatty liver disease: pathologic patterns and biopsy evaluation in clinical research. Semin Liver Dis.

[50] Kleiner DE, Brunt EM, Van Natta M, Behling C, Contos MJ (2005). Design and validation of a histological scoring system for nonalcoholic fatty liver disease. Hepatology.

[51] Setshedi M, Longato L, Petersen DR, Ronis M, Chen WC (2011). Limited Therapeutic Effect of N-Acetylcysteine on Hepatic Insulin Resistance in an Experimental Model of Alcohol-Induced Steatohepatitis. Alcohol Clin Exp Res.

[52] Stepanov I, Hecht SS (2005). Tobacco-specific nitrosamines and their pyridine-N-glucuronides in the urine of smokers and smokeless tobacco users. Cancer Epidemiol Biomarkers Prev.

[53] Stepanov I, Hecht SS (2008). Detection and quantitation of N′-nitrosonornicotine in human toenails by liquid chromatography-electrospray ionization-tandem mass spectrometry. Cancer Epidemiol Biomarkers Prev.

[54] Tricker AR, Ditrich C, Preussmann R (1991). N-nitroso compounds in cigarette tobacco and their occurrence in mainstream tobacco smoke. Carcinogenesis.

[55] de la Monte SM, Tong M (2009). Mechanisms of nitrosamine-mediated neurodegeneration: potential relevance to sporadic Alzheimer’s disease. J Alzheimers Dis.

[56] de la Monte SM, Tong M, Lawton M, Longato L (2009). Nitrosamine exposure exacerbates high fat diet-mediated type 2 diabetes mellitus, non-alcoholic steatohepatitis, and neurodegeneration with cognitive impairment. Mol Neurodegener.

[57] March TH, Wilder JA, Esparza DC, Cossey PY, Blair LF (2006). Modulators of cigarette smoke-induced pulmonary emphysema in A/J mice. Toxicol Sci.

[58] Upadhyaya P, Lindgren BR, Hecht SS (2009). Comparative levels of O6-methylguanine, pyridyloxobutyl-, and pyridylhydroxybutyl-DNA adducts in lung and liver of rats treated chronically with the tobacco-specific carcinogen 4-(methylnitrosamino)-1-(3-pyridyl)-1-butanone. Drug Metab Dispos.

[59] Trushin N, Rivenson A, Hecht SS (1994). Evidence supporting the role of DNA pyridyloxobutylation in rat nasal carcinogenesis by tobacco-specific nitrosamines. Cancer Res.

[60] Brunnemann KD, Rivenson A, Adams JD, Hecht SS, Hoffmann D (1987). A study of snuff carcinogenesis. IARC Sci Publ.

[61] CDC (1978). Occupational Health Guideline for Carbon Monoxide.

[62] Yu HS, Oyama T, Isse T, Kitagawa K, Pham TT (2010). Formation of acetaldehyde-derived DNA adducts due to alcohol exposure. Chem Biol Interact.

[63] Sun L, Luo C, Long J, Wei D, Liu J (2006). Acrolein is a mitochondrial toxin: effects on respiratory function and enzyme activities in isolated rat liver mitochondria. Mitochondrion.

